# Supplementary material for: Association of chronic neutrophil activation with risk of mortality
Source: PLoS One. 2023 Jul 20;18(7):e0288712. doi: 10.1371/journal.pone.0288712 (PMC10358907; doi:10.1371/journal.pone.0288712)
Supplement: Attachment — Submitted filename: review.docx [file pone.0288712.s002.docx]

**#comments**

Abstract

Participant- it is better if sampling method and statistical analysis was mentioned

Conclusion: it says, free circulating MPO is a strong marker of elevated risk of… ? In-complete sentence.

Method

Citation needed for sample size calculation

Which software did you used for data entry?

Did you check the model fitness?

Did you used log rank test for comparison, if so should stated

Result

Why don’t you present the age group in the table rather than repeating mean, already mentioned in narration?

Discussion

The discussion should structured as summarization of the result, it is better if arguing with previous studies. It shouldn’t repeat the result.
