# Supplementary material for: Association of chronic neutrophil activation with risk of mortality
Source: PLoS One. 2023 Jul 20;18(7):e0288712. doi: 10.1371/journal.pone.0288712 (PMC10358907; doi:10.1371/journal.pone.0288712)
Supplement: Attachment — Submitted filename: PONE-D-22-14255-R1 Response to Review 060623.docx [file pone.0288712.s003.docx]

We thank the Reviewers for their careful review of our manuscript. We have tried to address each of the concerns raised below.

**Abstract**

**Participant- it is better if sampling method and statistical analysis was mentioned**

As suggested, we have added the sampling method and statistical approach to the abstract in the revised submission.

**Conclusion: it says, free circulating MPO is a strong marker of elevated risk of… ? In-complete sentence.**

We thank the Reviewer for catching this. We have completed the sentence (added death) in the revised submission.

**Method**

**Citation needed for sample size calculation**

We have added references 17 and 18 that support the sample size calculations that were done

**Which software did you used for data entry?**

The CRO used a secure database that was based on Excel. This has been added to the revised submission.

**Did you check the model fitness? Did you use log rank test for comparison, if so should stated.**

We did and have edited the revised submission on pages 9 and 10 accordingly.

**Result**

**Why don’t you present the age group in the table rather than repeating mean, already mentioned in narration?**

The paragraph in the results the Reviewer is referring to summarizes Table 1. We feel it is important to highlight specific demographics in the results. We do not feel the repeating of means is inappropriate and Table 1 has more information including the difference in means between the groups and their standard deviations. Therefore, we, respectfully, have chosen not to alter Table 1.

**Discussion**

**The discussion should structured as summarization of the result, it is better if arguing with previous studies. It shouldn’t repeat the result.**

We understand and agree with the Reviewer. The discussion has been heavily edited and any restatements of the results of the study have been removed. We feel the discussion is tighter and flows better and thank the Reviewer for the suggestion.
